# The impact of hot nights on dengue incidence: a nationwide case crossover study in Brazil

**DOI:** 10.1186/s40249-025-01326-4

**Published:** 2025-06-16

**Authors:** Mintao Su, Junjun Chen, Zhisheng Liang, Qinfeng Zhou, Junxiong Ma, Huining Yang, Shaym Biswal, Murugappan Ramanathan, Haojun Fan, Fan Dai, Wei Huang, Minghui Ren, Zhenyu Zhang

**Affiliations:** 1 Department of Global Health, Peking University School of Public Health, 38 Xue Yuan Road, Haidian District, Beijing, 100191 China; 2 Department of Electrical and Computer Engineering, Johns Hopkins University, Baltimore, USA; 3 Department of Disease Prevention and Control, Third Medical Center of General Hospital of the People’s Liberation Army, Beijing, China; 4 Bloomberg School of Public Health, Johns Hopkins University, Baltimore, USA; 5 School of Medicine, Johns Hopkins University, Baltimore, USA; 6 Institute of Disaster and Emergency Medicine, Tianjin University, Tianjin, China; 7 California-China Climate Institute, University of California Berkeley School of Law, Berkeley, CA USA; 8 Institute of Environmental Medicine, Peking University School of Public Health, Beijing, 100191 China; 9 China Center for Health Development Studies, Peking University, Beijing, China; 10 Institute for Global Health and Development, Peking University, Beijing, China; 11 Institute of Carbon Neutrality, Peking University, Beijing, China

**Keywords:** Dengue, Hot nights, Temperature, Attributable risk, Brazil

## Abstract

**Background:**

Dengue fever, a mosquito-borne disease, is influenced by temperature. As global warming intensifies, the frequency of hot nights has increased. However, the relationship between hot nights and dengue transmission remains unclear. This study aimed to evaluate the impact of hot night exposures on dengue incidence.

**Methods:**

We collected individual dengue data from Brazil's SINAN database (2014–2021), covering 5,708,691 patients. Hot night exposures, including the average maximum nighttime temperatures, hot night excess, and hot night duration, were calculated using the ERA5-land dataset. A case-crossover design was employed to assess the association between each hot night exposure and dengue incidence using conditional logistic regression.

**Results:**

The average maximum nighttime temperature, hot night excess, and hot night duration were all significantly associated with increased risk of dengue, with odds ratios (*OR*s) of 1.86 (95% *CI* 1.86–1.87), 1.01 (95% *CI* 1.01–1.01), and 1.05 (95% *CI* 1.05–1.05), respectively. The attributable risks for each hot night exposure were 14.02% (95% *CI* 13.49%–14.60%), 27.80% (95% *CI* 27.33%–28.21%), and 26.95% (95% *CI* 26.58%–27.38%), respectively, when the exposure value was above the 90th percentile of its distribution.

**Conclusions:**

Hot night exposures were associated with an increased risk of dengue in Brazil. The public health burden of dengue is likely to rise with increasing hot night exposures, especially as hot nights become more frequent worldwide. Implementation of targeted vector control strategies and improved access to cooling equipment, such as air-conditioning, may serve as important mitigation measures.

**Graphical Abstract:**

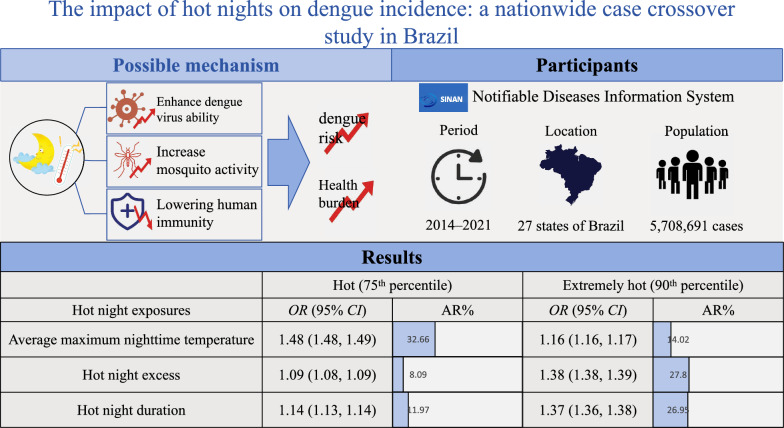

**Supplementary Information:**

The online version contains supplementary material available at 10.1186/s40249-025-01326-4.

## Background

Dengue fever, a primarily mosquito-borne viral infection, has emerged as a major concern, especially in tropical and subtropical regions [1]. The transmission of dengue closely follows the life cycle of mosquitoes, and is driven by a variety of factors, including socioeconomic class, latitude, and climatic conditions [2]. Among these, rising temperatures due to climate change are of increasing concern. Global warming has already contributed to an increase in dengue incidence and an expansion of transmission areas, especially in countries such as Brazil [3].

The recent surge in dengue cases is particularly concerning in regions such as Latin America and Asia, where an increasing number of people are being exposed to the virus [4]. Due to a variety of factors, dengue cases are distributed in Brazil from tropical to subtropical areas and from the wealthy urban areas to the favelas. In 2024, Brazil reported over 6.6 million suspected dengue cases, representing a more than 300% increase compared to 2023 [5]. Climatic factors have played a significant role in the sharp increase in dengue cases. This severe dengue epidemic has imposed a significant health burden and caused economic losses in Brazil. In light of this situation, it is essential to examine the drivers of dengue transmission, particularly environmental factors.

Temperature is a critical environmental factor that affects mosquito populations and dengue transmission. Previous studies have demonstrated significant associations between increases in mean, maximum, and minimum temperatures and higher dengue incidence [6]. However, some studies have also suggested that extremely high local temperatures may inhibit mosquito growth [7], indicating a complex relationship between temperature and dengue risk.

While previous studies have primarily focused on the relationship between daytime temperature and dengue transmission, the role of hot nights remains underexplored. This is especially important as the activity of the *Aedes aegypti* mosquitoes, the primary vector of dengue, has been observed to extend into the night [8, 9]. Moreover, nighttime temperatures are rising more rapidly than daytime temperatures globally, leading to an increase in the frequency and intensity of hot nights [10]. Despite this emerging trend, few studies have examined how hot night exposures influence dengue risk, making it a timely and urgent issue for investigation.

Given these trends, exploring the relationship between hot night exposures and dengue risk has important public health implications for dengue prevention and control. In this study, by using an individual-level case-crossover control design, we aimed to evaluate the impact of hot night exposures on dengue morbidity and to identify vulnerable subpopulations and symptoms.

## Methods

### Study population and design

We obtained individual-level dengue case data from Brazil’s Information System of Notifiable Diseases (SINAN) [11]. The SINAN database is anonymized and contains detailed information on confirmed and suspected dengue cases, including age, sex, race, education, state of residence, date of diagnosis, clinical symptoms (fever, myalgia, headache, rash, vomiting, nausea, back pain, conjunctivitis, arthritis, arthralgia, petechiae, positive tourniquet test, and retroorbital pain), and final diagnoses. Between 2014 and 2021, SINAN recorded a total of 11,427,494 cases or suspected cases of dengue. Final diagnoses were determined through laboratory confirmation or clinical evaluation [12]. We excluded records without a final diagnosis of dengue, dengue with warning signs, or severe dengue (*n* = 5,659,070), as well as cases outside the study period (2014–2021; *n* = 3,430), and those with missing age or date of birth (*n* = 56,303). After these exclusions, a total of 5,708,691 dengue cases were included in the final analysis (sFigure 1).

We conducted a case-crossover study to assess the association between hot night exposures and dengue incidence. This self-matching design is widely used in environmental epidemiology to assess the effects of environmental exposures [13]. By comparing exposure levels prior to disease onset with those on matched control days, this design effectively controls for seasonal variation and time-invariant or slowly changing confounders, such as sex, race, and underlying health status. Considering dengue's incubation period of 1–14 days, we defined the case period as the days prior to the diagnosis date and selected three reference control periods corresponding to the same calendar day in previous years within the same month (sFigure 2).

### Hot night exposures assessment

We obtained temperature data from the ERA5-Land dataset produced by the European Centre for Medium-Range Weather Forecasts [14]. This dataset has a spatial resolution of 0.1° × 0.1° and a temporal resolution of one hour. We obtained temperature data at 2 m above the Earth's land surface and extracted hourly average temperatures for 27 Brazilian states. We calculated the state-level average maximum nighttime temperatures, hot night excess (HNe), and hot night duration index (HNd) based on the state of residence and the date of diagnosis to assess hot night exposures for each case and control. HNe represents the sum of the excess nighttime temperatures above the threshold temperature (Tthr), while HNd quantifies the excess duration as a percentage of total nighttime hours. Tthr was defined as the 95th percentile of the minimum nighttime temperatures within a moving window of three months (90 days) before the date of diagnosis. We calculated hot night exposures for periods of 1, 2, and 3 months prior to the date of diagnosis. Detailed definitions and calculations are described in the supplementary material (sMethod 1). The night was defined as the period between local sunset and sunrise, and calculated using the centroid of each Brazilian state. The calculation was performed using the maptools package in R 4.3.3 (Lucent Technologies, Jasmine Mountain, USA), which utilizes the National Oceanic and Atmospheric Administration algorithm to determine local sunset and sunrise times. These times were then converted to Coordinated Universal Time to ensure consistency with the hourly temperature data from the ERA5 dataset.

### Covariates and analysis

Although the case-crossover study design minimized the effect of confounding factors, some factors may have changed due to the one-year interval between cases and controls. To further control for potential confounders, we obtained some covariates, including nighttime relative humidity (RH) (sMethod 2), risk factors, and sociodemographic and socioeconomic indicators.

State-level risk factor data were obtained from the Global Burden of Disease database [15]. We extracted disability-adjusted life years (DALYs) of all-causes for all level 2 risk factors, stratified by age group (0 to 4, 5 to 9, 10 to 14, …, 85 to 89, 90 to 94, and 95+ years) and sex (male and female) across each Brazilian state. Further details are provided in the supplementary material (sMethod 3). Sociodemographic and socioeconomic data at the state level were sourced from the Brazilian Institute of Geography and Statistics (IBGE) [16]. These included gross domestic product (trillion R$), population (millions), unemployment rate (%), crude mortality rate (‰), net migration rate (‰), and infant mortality rate (‰). We also collected data on life expectancy by age and sex. Latitude data for each state were also obtained from IBGE and categorized into 0° to 10°, 10° to 20°, and 20° to 30°.

We applied principal component analysis (PCA) to address multicollinearity and reduce the dimensionality of the risk factors and sociodemographic and socioeconomic indicators (sTable 1). This analysis provided a low-dimensional representation of these variables (sFigure 3B, 3C, and 3D). The component scores illustrated the correlations between specific variables and the different principal components (PCs) (sFigure 4). Details of each PC are in the supplementary material (sMethod 4). We retained PC1, PC2, PC3, and PC4, which explained over 80% of the cumulative variance (sFigure 3A).

### Statistical analysis

Variables were summarized as counts (*n*) and percentages (%) for categorical variables and as means and standard deviations (*SD*s) for continuous variables. Differences between dengue cases and controls were evaluated using the *t*-test.

We estimated the *OR*s of dengue incidence associated with each 1 ℃ increase in average maximum nighttime temperature, each 10 ℃ increases in HNe, and each 10% increase in HNd. Conditional logistic regression was performed, adjusting for nighttime RH and PCA terms, with stratification by matched sets of each dengue case and its corresponding controls. We estimated *OR*s for exposure periods of 1, 2, and 3 months, with a focus on the 1-month exposure period to capture short-term effects. The model included an interaction term between hot night exposures and region (Brazilian states) to capture geographic variability.

Subgroup analyses evaluated the association between hot night exposures and dengue incidence across different categories, including sex (male and female), age (0 to 17, 18 to 65, 66 to 79, and over 80 years), race (white, black, brown, Indigenous, and yellow), education (no education to incomplete middle school; complete middle school to incomplete high school; complete high school to incomplete higher education; and complete higher education), latitude (0° to 10°, 10° to 20°, and 20° to 30°), and nighttime RH (0% to 75%, 75% to 85%, and 85% to 100%). This was achieved by including multiplicative interaction terms between hot night exposures and each variable. We also assessed the association between hot night exposures and clinical symptoms of dengue by performing conditional logistic regression stratified by clinical symptoms.

To reveal the exposure–response relationship between hot night exposures and dengue incidence, we used a restricted cubic spline (RCS) function. Knots were selected based on the minimum Akaike Information Criterion (AIC). The final RCS curves for each hot night exposure included seven knots placed at the 2.50th, 18.33rd, 34.17th, 50.00th, 65.83rd, 81.67th, and 97.50th percentiles.

To assess the burden of dengue attributed to hot night exposures, we categorized hot night exposures into binary variables using different cutoff values based on three assumptions. In assumptions 1 and 2, based on values used in previous studies [17], the cutoffs were set as the 75th and 90th percentiles of hot night exposures to represent hot and extremely hot conditions, respectively. In assumption 3, we calculated different cutoff values for each of the 12 months of the year for each state by averaging the hot night exposures for that month over the period from 2011 to 2021. Attributable risk (AR%) and population-attributable risk (PAR%) were calculated using equations described in the supplementary material (sMethod 5).

We also conducted sensitivity analysis to demonstrate the robustness of the results. First, we excluded dengue cases diagnosed on or after January 1, 2020, to avoid the potential impact of COVID-19 outbreak prevention and control measures on dengue incidence. Second, we excluded dengue cases from São Paulo and Minas Gerais, totaling nearly 3 million cases, in order to avoid possible skews in the dataset that could be caused by the disproportionately large national share of dengue cases in these two states. Third, we defined night as the period from 0:00 a.m. to 8:00 a.m. the next morning local time to describe the time when people usually sleep. Fourth, we included an additional future control by selecting the same calendar day in the following year, in addition to the two past controls on the same day of the previous years. Fifth, we selected control days by matching on the same day of the week within the same quarter and year as the diagnosis date. In addition, the date of dengue diagnosis and its control dates were separated from each other by 4 weeks. As a result, each case could be matched with 2 or 3 controls. The fourth and fifth sensitivity analyses could help address the confounding bias introduced by the long-term temporal trends in temperature and dengue. Sixth, the Tthr was alternatively defined as the 95th percentile of minimum nighttime temperatures from 1990 to 2021 in each state to examine the robustness of our primary threshold selection (sMethod 1).

All associations were reported with corresponding 95% confidence intervals (*CI*s), including *OR*s, AR% and PAR%. A two-sided *P*-value < 0.05 was considered statistically significant. Statistical analyses were performed using R 4.3.3 (Lucent Technologies, Jasmine Mountain, USA), and mapping was conducted with ArcGIS 10.7 (Environmental Systems Research Institute, RedLands, USA).

## Results

### Descriptive results

Our study included a total of 5,708,691 dengue patients (mean age: 34.81 years; 2,547,885 males [44.6%]) (Table 1). Most cases were identified as brown (31.3%) or white (34.5%). The most common symptoms among dengue cases were fever (50.8%), myalgia (46.7%), and headache (47.3%). Notably, 37.6% of dengue patients had missing data on their clinical symptoms. During the 1-month exposure period, the average maximum nighttime temperature was 25.45 °C, the HNe was 307.47 °C, and the HNd was 42.78% (Table 2). Nighttime RH and average maximum nighttime temperature were significantly lower in the control group across all exposure periods, while HNe and HNd were significantly higher.

**Table 1:** Characteristics of dengue cases in Brazil, 2014–2021

Variables	Dengue case (*n* = 5,708,691)
Age, years	34.81 (18.86)
Age group	
0 to 17	1,130,270 (19.8)
18 to 65	4,202,138 (73.6)
66 to 79	309,782 (5.4)
Over 80	66,501 (1.2)
Sex	
Male	2,547,885 (44.6)
Female	3,160,806 (55.4)
Race	
White	1,967,102 (34.5)
Black	200,358 (3.5)
Brown	1,787,229 (31.3)
Indigenous	12,050 (0.2)
Yellow	40,657 (0.7)
Missing	1,701,295 (29.8)
Education	
No education—incomplete middle school	744,016 (13.0)
Complete middle school—incomplete high school	526,680 (9.2)
Complete high school—incomplete higher education	850,081 (14.9)
Complete higher education	206,009 (3.6)
Missing	3,381,905 (59.2)
Latitude	
0° to 10°	825,848 (14.5)
10° to 20°	2,275,041 (39.9)
20° to 30°	2,607,802 (45.7)
Symptoms	
Fever	2,900,748 (50.8)
Myalgia	2,668,496 (46.7)
Headache	2,701,621 (47.3)
Rash	859,606 (15.1)
Vomiting	741,807 (13.0)
Nausea	1,206,321 (21.1)
Backpain	1,006,968 (17.6)
Conjunctivitis	126,196 (2.2)
Arthritis	395,253 (6.9)
Arthralgia	745,317 (13.1)
Petechiae	378,571 (6.6)
Positive tourniquet test	176,356 (3.1)
Retroorbital pain	1,244,430 (21.8)

Values are mean (*SD*, standard deviation) or *n* (%)

**Table 2:** Hot night exposures and nighttime relative humidity exposure of participants

Variables	Case (*n* = 5,708,691)	Control (*n* = 17,123,073)	*P*
Average maximum nighttime temperature, ℃			
1 month	25.45 (1.87)	25.26 (1.97)	< 0.001
2 months	25.58 (1.65)	25.41 (1.74)	< 0.001
3 months	25.71 (1.54)	25.47 (1.61)	< 0.001
Hot night excess, ℃			
1 month	307.47 (201.45)	320.60 (199.13)	< 0.001
2 months	652.05 (326.50)	682.89 (325.71)	< 0.001
3 months	1,046.96 (408.30)	1,059.13 (414.03)	< 0.001
Hot night duration, %			
1 month	42.78 (18.70)	44.46 (18.96)	< 0.001
2 months	46.00 (14.70)	47.74 (14.74)	< 0.001
3 months	49.01 (11.76)	49.72 (11.65)	< 0.001
Nighttime relative humidity, %			
1 month	80.71 (8.28)	80.34 (8.37)	< 0.001
2 months	81.07 (7.51)	80.60 (7.68)	< 0.001
3 months	80.75 (7.11)	80.60 (7.26)	< 0.001

Note: Values are mean (*SD*, standard deviation). *P* values were calculated using the *t*-test

### Association between hot night exposures and dengue incidence

**Fig. 1:**
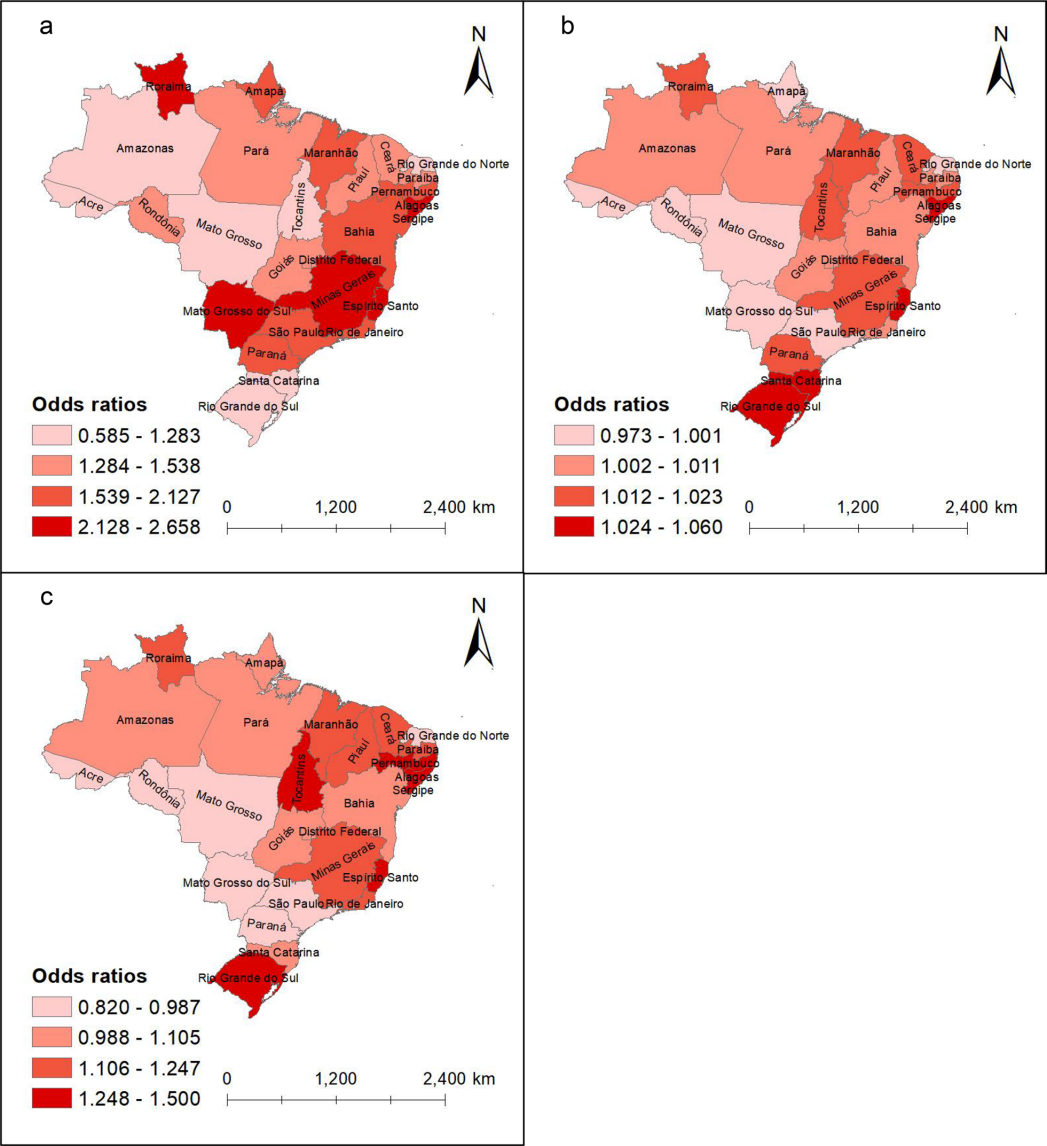
Distribution of the association between hot night exposures and dengue incidence in Brazilian states by quartile. **a** Average maximum nighttime temperature; **b** Hot night excess; **c** Hot night duration. The shade of color represents the odds ratio (*OR*) of the association between dengue risk and hot night exposures in the state. Lighter shades of red indicate lower *OR* values, while darker shades signify higher *OR* values. The basemap for the maps was sourced from Natural Earth, and the download link is: https://www.naturalearthdata.com/downloads/10m-cultural-vectors/, which is under a CC0 (public domain) license (http://www.naturalearthdata.com/about/terms-of-use/)

In the main analysis, we found that during the 1-month exposure period, increasing hot night exposures were associated with higher risks of dengue (Table 3 and sTable 2). Each 1 ℃ increase in average maximum nighttime temperature in Brazil was associated with an *OR* of 1.86 (95% *CI* 1.86–1.87) for dengue incidence. Both the intensity and duration of hot nights were positively associated with dengue, with an *OR* of 1.01 (95% *CI* 1.01–1.01) per 10 ℃ increases in HNe and an *OR* of 1.05 (95% *CI* 1.05–1.05) per 10% increase in HNd. The association remained consistent across 2- and 3-months exposure periods. Average maximum nighttime temperature had a stronger association with dengue incidence in southern Brazil, such as Mato Grosso do Sul (*OR* = 2.23, 95% *CI* 2.21–2.25) and Minas Gerais (*OR* = 2.50, 95% *CI* 2.49–2.51). HNe and HNd showed stronger associations with dengue incidence in the eastern and southern states, such as Alagoas (*OR* = 1.04, 95% *CI* 1.04–1.04; *OR* = 1.35, 95% *CI* 1.34–1.37) and Rio Grande do Sul (*OR* = 1.03, 95% *CI* 1.01–1.01; *OR* = 1.39, 95% *CI* 1.35–1.43) (Fig. 1 and sTable 2).

**Table 3:** Association between hot night exposures and dengue incidence

Exposures	*OR* (95% *CI*)		
	1 month	2 months	3 months
Average maximum nighttime temperature, ℃	1.86 (1.86, 1.87)	1.83 (1.83, 1.84)	2.03 (2.03, 2.04)
Hot night excess, 10 ℃	1.01 (1.01, 1.01)	1.00 (1.00, 1.00)	1.01 (1.01, 1.01)
Hot night duration, 10%	1.05 (1.05, 1.05)	1.00 (1.00, 1.00)	1.14 (1.13, 1.14)

The model was adjusted for nighttime relative humidity, PC1, PC2, PC3, and PC4. OR: Odd ratio, CI: Confidence interval

The association between average maximum nighttime temperature, HNd, and dengue incidence were higher in males (sFigure 5). Additionally, the association between average nighttime maximum temperature and dengue risk was higher in the elderly, whereas HNd and HNe did not show a harmful effect on the risk of dengue. The association between dengue incidence and average maximum nighttime temperature was stronger at higher latitudes, such as in the 20° to 30° range (*OR* = 1.89, 95% *CI* 1.89–1.90). Dengue risk increased with higher humidity levels, with the strongest association between average nighttime maximum temperature and dengue risk observed at high nighttime RH (85% to 100%), where the *OR* was 2.17 (95% *CI* 2.16–2.18). Among clinical symptoms, hot nights increased the risk of vomiting, with a 72% increase (*OR* = 1.72, 95% *CI* 1.71–1.72) per 1 ℃ increase in the average maximum nighttime temperature, which was higher than the 69% increase (*OR* = 1.69, 95% *CI* 1.69–1.70) observed in patients without vomiting (sFigure 6).

**Fig. 2:**
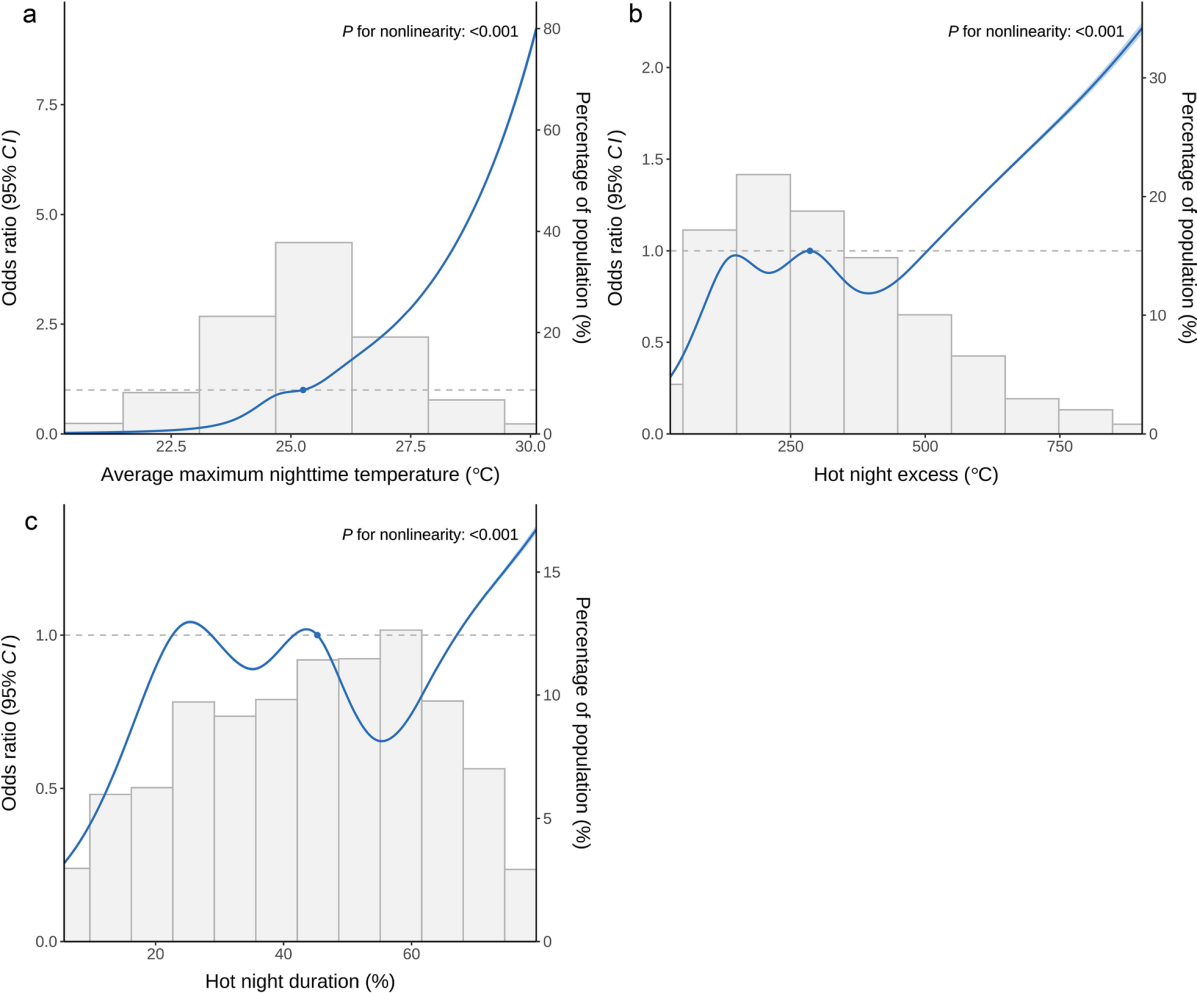
Exposure–response curve between hot night exposures and dengue incidence. **a** Average maximum nighttime temperature. **b** Hot night excess. **c** Hot night duration. The exposure–response curve was calculated using restricted cubic splines with knots at the 2.50th, 18.33rd, 34.17th, 50.00th, 65.83rd, 81.67th, and 97.50th percentiles of the distribution of exposures. The reference exposure level was set at the 50th percentile of the distribution of average maximum nighttime temperature (25.25 ℃), hot night excess (285.81 ℃), and hot night duration (45.28%). Odds ratios were adjusted for nighttime relative humidity, PC1, PC2, PC3, PC4. The solid line indicates the estimated odds ratio values, and the dashed lines indicate their 95% confidence intervals. The bars are histograms (dependent on the right y-axis) and indicate the distribution of the corresponding exposure level. The blue dot represents an odds ratio of 1, indicating the reference level for the corresponding exposure

The association between average maximum nighttime temperature and dengue incidence increased with rising temperature (Fig. 2). HNe and HNd showed local fluctuations and an overall increasing trend. All three hot night exposures exhibited non-linear associations with dengue incidence.

We assessed the dengue burden attributed to hot night exposures (Table 4). At the 75th percentile cutoff, the AR% of dengue incidence associated with average maximum nighttime temperature, HNe, and HNd was 32.66% (95% *CI* 32.30%–32.98%), 8.09% (95% *CI* 7.66%–8.51%), and 11.97% (95% *CI* 11.58%–12.28%), respectively. The PAR% was 10.43% (95% *CI* 10.27%–10.56%), 2.24% (95% *CI* 2.11%–2.36%), and 3.42% (95% *CI* 3.29%–3.51%), respectively. When the cutoff was set at the 90th percentile, representing extremely hot nights, the AR% and PAR% for HNe and HNd increased, while those for average maximum nighttime temperatures decreased. Under assumption 3, the AR% and PAR% varied but did not contradict the previous two assumptions.

**Table 4:** AR% and PAR% for dengue using different hot night exposures in Brazil

	Controls exposed above the cutoff/Total controls	*OR* (95% *CI*)	AR%	PAR%
Assumption 1				
Average maximum nighttime temperature	4,083,340/17,126,073	1.48 (1.48, 1.49)	32.66 (32.30, 32.98)	10.43 (10.27, 10.56)
Hot night excess	4,407,491/17,126,073	1.09 (1.08, 1.09)	8.09 (7.66, 8.51)	2.24 (2.11, 2.36)
Hot night duration	4,454,657/17,126,073	1.14 (1.13, 1.14)	11.97 (11.58, 12.28)	3.42 (3.29, 3.51)
Assumption 2				
Average maximum nighttime temperature	1,705,759/17,126,073	1.16 (1.16, 1.17)	14.02 (13.49, 14.60)	1.60 (1.54, 1.68)
Hot night excess	1,729,866/17,126,073	1.38 (1.38, 1.39)	27.80 (27.33, 28.21)	3.71 (3.62, 3.78)
Hot night duration	1,715,154/17,126,073	1.37 (1.36, 1.38)	26.95 (26.58, 27.38)	3.56 (3.49, 3.63)
Assumption 3				
Average maximum nighttime temperature	10,049,011/17,126,073	1.57 (1.57, 1.58)	36.39 (36.14, 36.59)	25.23 (25.03, 25.4)
Hot night excess	9,350,562/17,126,073	1.25 (1.25, 1.25)	20.00 (19.74, 20.26)	12.09 (11.92, 12.26)
Hot night duration	10,879,162/17,126,073	1.12 (1.12, 1.13)	10.87 (10.63, 11.11)	7.24 (7.08, 7.41)

AR%: attributable risk. PAR%: population attributable risk

In assumption 1, the cutoff value was taken as the 75th percentile of each hot night exposure (26.47 ℃ for average maximum nighttime temperature, 433.82 ℃ for hot night excess, and 58.89% for hot night duration). In assumption 2, the cutoff value was taken as the 90th percentile of each hot night exposure (27.72 ℃ for average maximum nighttime temperature, 589.78 ℃ for hot night excess, and 68.44% for hot night duration). In assumption 3, we calculated different cutoff values for each of the 12 months of the year for each state by averaging the hot night exposures for that month over the period from 2011 to 2021. For example, Acre's average maximum nighttime temperature cutoff value for January was determined by calculating the average of the daily maximum nighttime temperatures for January across the years 2011 to 2021. The model was adjusted for nighttime relative humidity, PC1, PC2, PC3, and PC4

### Sensitivity analysis results

Multiple sensitivity analyses exhibited similar results to the main analysis, indicating the robustness of the findings (sTable 4).

## Discussion

This study found that hot night exposures were associated with an increased risk of dengue. These associations varied across Brazilian states, with males, the elderly, and those living in high-latitude areas or regions with high RH identified as particularly vulnerable. Vomiting was a clinical symptom that was prone to occur due to the influence of hot night exposures. As hot nights become more frequent, the intensity and duration of nighttime heat will play a critical role in influencing dengue risk.

Our findings suggest that hot night exposures contribute to dengue transmission. Mechanistically, both global climate change and local factors, such as land use change and the urban heat island effect, may further increase nighttime temperatures, leading to more hot nights. Hot nights may enhance dengue virus activity in mosquitoes. Laboratory studies have demonstrated that elevated temperatures increase dengue virus replication in the mosquitoes and shorten the extrinsic incubation period of the virus [18]. Moreover, rising nighttime temperatures may expand the geographic range suitable for dengue mosquito vectors. The maximum nighttime temperatures observed in this study (typically 24–27 °C) fall within the optimal range for vector survival [19], which has been shown to increase mosquito fertility, longevity, and biting rate. Previous studies have reported that dengue transmission by *Aedes aegypti* increased by 28% from 1951–1960 to 2013–2022, driven by rising temperatures, and is expected to continue increasing in the future [20].

The variation in dengue incidence risk among Brazilian states and latitude ranges may be attributed to local conditions and geographic factors [21]. In high-risk areas, dengue cases often coincide with higher hot night exposures compared to controls. In addition, previous studies have found that the distribution of mosquitoes decreases with increasing latitude in high-latitude areas of Brazil, and the number of dengue cases exhibits a similar pattern [22]. However, the increases in hot night exposures may expand the range of mosquito distribution to higher latitudes, leading to a rise in dengue incidence [23]. These findings highlight the need for greater regional attention to the dengue risk posed by hot night exposures. For instance, the central-southern and high-latitude regions should focus on managing maximum nighttime temperature, while the southernmost regions should consider the impact of sustained high nighttime temperatures.

Our study also found that males and the elderly are particularly vulnerable to dengue, consistent with findings from previous studies [24]. While *Aedes aegypti* primarily rests and bites indoors, its biting activity can also occur in outdoor or semi-open environments [25]. In settings with limited protective infrastructure, males, who are more likely to be exposed to outdoor environments during work or commuting, may face higher exposure risk. However, some studies contradict this conclusion, suggesting that female morbidity risk is higher, which may be due to a higher tendency for females to actively seek medical care than males, thus contributing to the morbidity bias [26]. Some studies have identified the elderly as a high-risk group due to age-related decline in immune function [27]. Children may also be vulnerable, as adults tend to accumulate multitypic immunity over time [28], leaving younger populations more susceptible to primary infections. Higher nighttime RH may contribute to an increased risk of dengue transmission, potentially because low humidity accelerates water loss from mosquitoes through evaporation, thereby reducing their survival efficiency [29]. In addition, the substantial amount of missing data on race and education in this study may have introduced bias. More research is needed to explore high-risk groups and underlying mechanisms.

Vomiting is a clinical symptom of dengue fever that appears to be sensitive to hot night exposures. There are few previous studies on the factors influencing the clinical symptom of dengue fever, and the mechanisms involved remain unclear. This may be related to heat-induced gastrointestinal dysfunction caused by thermal stress and other physiological responses in hot environments, which may manifest as vomiting. The potential impact on other clinical symptoms should also be considered. Healthcare providers should ensure the availability of appropriate symptomatic medications during hot nights.

Unlike the inverted U-shaped relationship between daily temperatures and dengue incidence reported in previous studies [30], our findings indicated a generally nonlinear increase in dengue risk with rising hot night exposures. This pattern may be explained by the fact that nighttime temperatures in Brazil have not yet reached thresholds that inhibit mosquito growth and activity [19]. According to this exposure–response relationship, the risk of dengue incidence may continue to rise as global warming drives further increases in nighttime temperatures. This finding highlights the importance of focusing on the impact of hot nights on dengue transmission. One potential protective measure is the use of air-conditioning, which may reduce dengue risk by reducing the abundance of *Aedes* mosquitoes indoors [31].

The risk of dengue attributed to hot night exposures is significant and should not be overlooked. Individuals should be aware of extreme and prolonged nighttime heat, as over 20% of dengue risk is attributable to the intensity and duration of heat during extremely hot nights. Previous studies have suggested that temperature is responsible for many dengue cases, especially in large-scale outbreaks [32]. Our study underscores the need to implement and strengthen more targeted policies and interventions. In October 2024, the World Health Organization launched the Global Strategy for Prevention, Preparedness, and Response, which emphasizes that disease surveillance, laboratory activities, vector control, community engagement, clinical management, and research and development are all essential components in the fight against dengue. Our study highlights the important role of hot nights in dengue risk, providing scientific evidence to support vector control measures, prevention strategies, and policy development.

Our study has several strengths. We employed a case-crossover design and collected comprehensive covariate data to minimize potential confounders. This study represents the first systematic assessment of the association between hot night exposures and dengue incidence and symptoms. However, there are limitations to consider. First, while our data encompass all of Brazil, representing a significant portion of South America, caution is needed when extrapolating the results to other regions. Second, The SINAN database, despite its extensive coverage, may underreport cases, especially asymptomatic infections. Third, exposure assessments were based on state-level average temperatures, which may introduce measurement bias due to regional variability within states. State averages provide only a rough approximation of true exposure levels. Future studies should incorporate finer-resolution data for more accurate exposure assessments. Fourth, entomological surveillance data on mosquito vector presence or density were not available. As such, we were unable to directly assess the mediating role of vector abundance. Fifth, although we observed associations between hot night exposures and dengue incidence, the study design does not allow us to infer causality.

## Conclusions

This national study highlights the substantial burden that hot nights, particularly its intensity and duration, pose on dengue risk. As the intensity and frequency of hot nights continue to increase due to climate change and global warming, greater attention should be paid to the dengue risk associated with hot nights. Our study provides scientific evidence to support dengue control strategies, including vector management and nighttime heat mitigation.

## Supplementary Information

Supplementary material 1.

## Data Availability

The dengue cases dataset is available without restriction from the Notifiable Diseases Information System of Brazil (https://datasus.saude.gov.br/transferencia-de-arquivos/#). Temperature data were sourced from the ERA5-Land datasets of the European Center for Medium-Range Weather Forecasts (https://cds.climate.copernicus.eu/datasets/reanalysis-era5-land?tab=overview). State-level risk factor data for Brazil were obtained from the Global Burden of Disease database (https://vizhub.healthdata.org/gbd-results/), while sociodemographic and socioeconomic data were obtained from the Brazilian Institute of Geography and Statistics (https://www.ibge.gov.br/en/statistics/full-list-statistics.html). The shapefiles for the maps were sourced from Natural Earth (https://www.naturalearthdata.com/downloads/10m-cultural-vectors/). All data can be freely accessed and downloaded online from the respective public websites mentioned above.

## Abbreviations

SINAN: Information System of Notifiable Diseases
*O**R*: Odds ratio
*CI*: Confidence Interval
HNe: Hot night excess
HNd: Hot night duration
Tthr: Threshold temperature
RH: Relative humidity
IBGE: Brazilian Institute of Geography and Statistics
DALYs: Disability-adjusted life years
PCA: Principal component analysis
PC: Principal component
*S**D*: Standard deviation
RCS: Restricted cubic spline
AIC: Akaike Information Criterion
AR%: Attributable risk
PAR%: Population-attributable risk
