# Supplementary material for: The impact of hot nights on dengue incidence: a nationwide case crossover study in Brazil
Source: Infect Dis Poverty. 2025 Jun 16;14:50. doi: 10.1186/s40249-025-01326-4 (PMC12168315; doi:10.1186/s40249-025-01326-4)
Supplement: Supplementary file 1 — Supplementary material 1. [file 40249_2025_1326_MOESM1_ESM.docx]

Supplementary material for

**The impact of hot nights on dengue incidence: a nationwide case crossover study in Brazil**

Mintao Su, Junjun Chen, Zhisheng Liang, Qinfeng Zhou, Junxiong Ma, Huining Yang, Shaym Biswal, Murugappan Ramanathan, Haojun Fan, Fan Dai, Wei Huang, Minghui Ren, Zhenyu Zhang

**Context of the supplementary materials:**

1. **sMethod 1.** The definitions and calculations of hot night exposures.
2. **sMethod 2.** Definition and calculation of nighttime relative humidity.
3. **sMethod 3.** Risk factors inclusion and treatment.
4. **sMethod 4.** Description of each principal component (PC).
5. **sMethod 5.** Calculations of attributable risk and population attributable risk.
6. **sTable 1.** Variables used in the principal component analysis of participants.
7. **sTable 2.** Association between hot night exposures and dengue by Brazil state using conditional logistic regression.
8. **sTable 3.** Mean of hot night exposures by group and state.
9. **sTable 4.** Sensitivity analysis.
10. **sFigure 1.** The flowchart for the inclusion and exclusion of dengue patients.
11. **sFigure 2.** Case crossover design.
12. **sFigure 3.** Principal component analysis of the risk factor and socioeconomic data.
13. **sFigure 4.** Correlation coefficients between the variables and the PCs.
14. **sFigure 5.** Subgroup analysis for the association between hot night exposures and dengue incidence.
15. **sFigure 6.** Odds ratios of hot night exposures on dengue incidence with different clinical symptoms.
16. **References.**

**sMethod 1. The definitions and calculations of hot night exposures.**

The average maximum nighttime temperature (℃) is the average of the maximum temperatures for each night during the exposure period and is used to measure the level of maximum nighttime temperature. It was calculated using equation (Eq) (1).

$$\bar{T}_{max}=\frac{\sum_{i=1}^{N} T_{{max}_{i}}}{N} (1)$$

In Eq (1), $\bar{T}_{max}$is the average maximum nighttime temperature, and $T_{{max}_{i}}$ denotes the maximum temperature at night on day i out of the number of days N in the exposure period.

HNe (10 ℃), which allows the assessment of nocturnal thermal stress^1 2^, assesses the intensity of exposure to hot night temperatures. It is obtained by calculating the sum of nighttime temperatures above the threshold temperature (Tthr) as shown in Eq (2).

$$HNe= \frac{\sum_{i=1}^{N} \sum_{j=1}^{n_{i}} \left( t_{ij}-Tthr \right)\times I_{Tthr}\left( t_{ij} \right)}{10} (2)$$

$t_{ij}$ denotes the temperature at the hour j of the night time duration n on day i out of the number of days N in the exposure period. $I_{Tthr}$ is the function that determines whether $t_{ij}$ is no less than Tthr as follows:

$$I_{Tthr}\left( t_{ij} \right)=\left\{ \begin{aligned} 0 if t_{ij}<Tthr \\ 1 if t_{ij}\geq Tthr \end{aligned} \right.$$

HNd (%), obtained by calculating the proportion of hours when the nighttime temperature is not below the threshold temperature during the exposure period (Eq 3), described the duration of high nighttime temperatures^1 2^.

$$HNd=\frac{\sum_{i=1}^{N} \sum_{j=1}^{n_{i}} I_{Tthr}\left( t_{ij} \right)}{\sum_{i=1}^{N} n_{i}}\times100 (3)$$

As suggested in previous research^2 3^, Tthr was obtained from hourly nighttime temperature data to quantify the hot night exposures. Nighttime temperatures exceeding Tthr will be classified as hot and included in the calculation of HNe and HNd using the equations described above. Considering the seasonal variation in temperature and the potential adaptability of organisms, including humans and mosquito, to temperature changes^4 5^, we calculate Tthr using a moving window for each dengue patient and their controls. Tthr was defined as the 95^th^ percentile of the minimum nighttime temperature in the moving window of three months (90 days) before the date of diagnosis (control date for controls).

**sMethod 2. Definition and calculation of nighttime** **relative humidity.**

Nighttime relative humidity (RH) is defined as the ratio of partial water vapor pressure to the saturation vapor pressure at a given temperature. We obtained dewpoint temperatures at 2 meters above the Earth's surface from the ERA5-land dataset and calculated hourly dewpoint temperatures for each Brazilian state. Using these hourly dewpoint temperatures and air temperatures obtained in the exposure assessment, we calculated average nighttime RH values for each exposure period with the Clausius-Clapeyron equation^6 7^ (Eq 4). We calculated nighttime RH values for periods of 1, 2, and 3 months prior to diagnosis of dengue. Nighttime is defined as the period between local sunset and sunrise, calculated using the National Oceanic and Atmospheric Administration (NOAA) algorithm.

$$RH=\frac{E_{0}\times exp\left( \frac{L}{R_{v}}\times\left( \frac{1}{T_{0}}-\frac{1}{T_{d}} \right) \right)}{E_{0}\times exp\left( \frac{L}{R_{v}}\times\left( \frac{1}{T_{0}}-\frac{1}{T_{a}} \right) \right)} (4)$$

Where $E_{0}=0.611$ is the saturated vapor pressure at reference temperature $T_{0}=273K$. $L=2.5\times{10}^{6}J\cdot{kg}^{-1}$ is the latent heat of evaporation for water. $R_{v}=461.52J\cdot{kg}^{-1}\cdot K^{-1}$ is the specific gas constant for water vapor. $T_{d}$ is the dewpoint temperature in Kelvin, while $T_{a}$ is the air temperature in Kelvin.

**sMethod 3. Risk factors inclusion and treatment.**

There are 20 age strata (0 to 4, 5 to 9, 10 to 14, …, 85 to 89, 90 to 94, and 95 + years) and 2 gender strata (male and female) for a total of 20 risk factors (level 2), including non-optimal temperature, tobacco, kidney dysfunction, drug use, high low-density lipoprotein (LDL) cholesterol, alcohol use, high body mass index (BMI), high fasting plasma glucose, childhood maltreatment, high systolic blood pressure, dietary risks, unsafe sex, occupational risks, unsafe water sanitation and handwashing, low physical activity, other environmental risks, air pollution, child and maternal malnutrition, intimate partner violence, and low bone mineral density. Intimate partner violence and low bone mineral density were excluded due to a high number of missing values. Other risk factors with low missingness were imputed with data from the nearest age group.

**sMethod 4. Description of each principal component (PC).**

PC1 mainly represents physical condition, with contributions including high low density lipoprotein cholesterol, high body mass index and high fast plasma glucose **(sFigure 4)**. PC2 is mainly associated with child maltreatment and represents childhood experiences. PC3 stands for hygiene and nutritional status and is mainly related to malnutrition and unsafe water sanitation and handwashing. PC4 mainly represents crude mortality rate.

**sMethod 5. Calculations of attributable risk and population attributable risk.**

The *OR*s obtained were then used to calculate the attributable risk (AR%) level and the population attributable risk (PAR%) level using Eq 5 and Eq 6 for each hot night exposure^8^.

$$AR\%=\frac{OR-1}{OR}\times100 (5)$$

$$PAR\%=\frac{p_{p}\times\left( OR-1 \right)}{p_{p}\times\left( OR-1 \right)+1} (6)$$

Where $p_{p}$ indicates the percentage of the total population exposed to each hot night exposure, which was approximated using the exposure rate of the control group in this study.

**sTable 1. Variables used in the principal component analysis of participants.**

| Characteristics | Case  (*n* = 5,708,691) | Control  (*n* = 17,126,073) | p |
| --- | --- | --- | --- |
| GBD risk factors |  |  |  |
| Non-optimal temperature | 642.80 (1,626.47) | 540.13 (1,630.23) | <0.001 |
| Tobacco | 10,116.67 (18,771.16) | 9,376.86 (17,891.40) | <0.001 |
| Kidney dysfunction | 3,797.66 (5,956.63) | 3,420.56 (5,471.55) | <0.001 |
| Drug use | 2,732.65 (3,409.17) | 2,403.55 (3,004.85) | <0.001 |
| High LDL cholesterol | 4,905.94 (8,624.31) | 4,428.88 (8,134.98) | <0.001 |
| Alcohol use | 9,103.33 (12,846.52) | 9,098.81 (13,238.02) | 0.476 |
| High body mass index | 10,447.88 (15,592.40) | 9,185.23 (14,187.26) | <0.001 |
| High fasting plasma glucose | 8,014.50 (13,147.65) | 6,944.81 (11,851.39) | <0.001 |
| Childhood maltreatment | 1,174.78 (1,306.92) | 1,189.01 (1,358.01) | <0.001 |
| High systolic blood pressure | 8,846.91 (16,278.26) | 7,931.15 (15,161.70) | <0.001 |
| Dietary risks | 7,621.95 (12,471.68) | 6,783.08 (11,574.38) | <0.001 |
| Unsafe sex | 3,392.35 (3,987.26) | 3,346.05 (4,125.79) | <0.001 |
| Occupational risks | 5,548.50 (5,875.94) | 5,391.08 (5,929.42) | <0.001 |
| Unsafe water sanitation and handwashing | 401.82 (536.75) | 466.69 (807.86) | <0.001 |
| Low physical activity | 908.53 (1,804.35) | 764.93 (1,595.23) | <0.001 |
| Other environmental risks | 984.08 (1,743.06) | 917.38 (1,667.88) | <0.001 |
| Air pollution | 3,661.02 (5,649.27) | 3,618.95 (5,569.54) | <0.001 |
| Child and maternal malnutrition | 6,611.05 (16,893.64) | 8,384.07 (22,876.94) | <0.001 |
| Sociodemographic and socioeconomic |  |  |  |
| GDP (trillion R$) | 0.92 (0.88) | 0.82 (0.78) | <0.001 |
| Population (millions) | 22.16 (16.83) | 21.79 (16.54) | <0.001 |
| Expectancy life (years) | 46.96 (16.84) | 48.33 (16.85) | <0.001 |
| Unemployment rate (%) | 0.11 (0.03) | 0.09 (0.03) | <0.001 |
| Crude mortality rate (‰) | 6.26 (0.59) | 6.18 (0.60) | <0.001 |
| Net migration rate (‰) | 0.85 (2.26) | 0.91 (2.39) | <0.001 |
| Infant mortality rate (‰) | 11.42 (2.87) | 12.29 (3.18) | <0.001 |

Abbreviations: GBD, Global Burden of Disease; LDL, low density lipoprotein; GDP, gross domestic product.

Note: GBD risk factors measures the all-cause disability life-adjusted years (DALYs) attributable to that risk factor. All variables are measured at the state level. Values are mean (*SD*). P values were calculated using the *t*-test.

**sTable 2. Association between hot night exposures and dengue by Brazil state using conditional logistic regression.**

|  |  | *OR* (95% *CI*) | | |
| --- | --- | --- | --- | --- |
|  | Cases (%) | Average maximum nighttime temperature | Hot night excess | Hot night duration |
| Brazil | 5,708,691 (100) | 1.864 (1.860, 1.868) | 1.010 (1.010, 1.010) | 1.047 (1.046, 1.049) |
| Acre | 71,725 (1.3) | 0.777 (0.761, 0.793) | 0.973 (0.972, 0.975) | 0.897 (0.887, 0.907) |
| Alagoas | 72,886 (1.3) | 2.658 (2.622, 2.695) | 1.037 (1.036, 1.038) | 1.354 (1.340, 1.369) |
| Amapá | 7,898 (0.1) | 2.115 (1.986, 2.253) | 0.998 (0.992, 1.004) | 1.022 (0.988, 1.057) |
| Amazonas | 24,977 (0.4) | 0.850 (0.822, 0.878) | 1.011 (1.009, 1.013) | 1.072 (1.056, 1.087) |
| Bahia | 147,485 (2.6) | 1.794 (1.778, 1.809) | 1.011 (1.010, 1.011) | 1.104 (1.096, 1.113) |
| Ceará | 219,017 (3.8) | 1.516 (1.504, 1.527) | 1.017 (1.016, 1.017) | 1.159 (1.153, 1.165) |
| Distrito Federal | 132,582 (2.3) | 1.373 (1.361, 1.385) | 1.008 (1.007, 1.008) | 1.064 (1.057, 1.072) |
| Espírito Santo | 162,774 (2.9) | 2.638 (2.616, 2.660) | 1.049 (1.048, 1.050) | 1.500 (1.490, 1.511) |
| Goiás | 585,748 (10.3) | 1.353 (1.346, 1.360) | 1.009 (1.008, 1.009) | 1.105 (1.101, 1.109) |
| Maranhão | 33,053 (0.6) | 1.951 (1.896, 2.006) | 1.023 (1.020, 1.025) | 1.190 (1.168, 1.213) |
| Mato Grosso | 108,111 (1.9) | 1.197 (1.182, 1.213) | 0.997 (0.996, 0.998) | 0.891 (0.882, 0.899) |
| Mato Grosso do Sul | 168,106 (2.9) | 2.229 (2.209, 2.249) | 0.994 (0.994, 0.995) | 0.820 (0.814, 0.825) |
| Minas Gerais | 1,104,622 (19.3) | 2.495 (2.486, 2.505) | 1.013 (1.012, 1.013) | 1.137 (1.134, 1.140) |
| Paraná | 420,075 (7.4) | 1.684 (1.675, 1.694) | 1.016 (1.015, 1.016) | 0.987 (0.982, 0.991) |
| Paraíba | 73,252 (1.3) | 1.392 (1.377, 1.406) | 1.005 (1.004, 1.005) | 1.113 (1.103, 1.124) |
| Pará | 26,809 (0.5) | 1.459 (1.421, 1.499) | 1.011 (1.008, 1.013) | 1.034 (1.016, 1.053) |
| Pernambuco | 165,618 (2.9) | 1.962 (1.947, 1.976) | 1.023 (1.022, 1.023) | 1.493 (1.482, 1.503) |
| Piauí | 32,116 (0.6) | 1.538 (1.514, 1.562) | 1.009 (1.008, 1.010) | 1.108 (1.096, 1.120) |
| Rio Grande do Norte | 48,320 (0.8) | 1.047 (1.032, 1.061) | 0.996 (0.995, 0.997) | 0.980 (0.970, 0.991) |
| Rio Grande do Sul | 18,714 (0.3) | 1.156 (1.133, 1.180) | 1.033 (1.029, 1.036) | 1.390 (1.353, 1.428) |
| Rio de Janeiro | 124,415 (2.2) | 2.127 (2.101, 2.153) | 1.011 (1.009, 1.012) | 1.146 (1.133, 1.159) |
| Rondônia | 16,610 (0.3) | 1.453 (1.403, 1.504) | 0.999 (0.996, 1.002) | 0.919 (0.897, 0.941) |
| Roraima | 4,465 (0.1) | 2.470 (2.288, 2.666) | 1.018 (1.012, 1.024) | 1.247 (1.190, 1.305) |
| Santa Catarina | 40,493 (0.7) | 0.585 (0.575, 0.594) | 1.026 (1.022, 1.029) | 1.103 (1.079, 1.127) |
| São Paulo | 1,835,999 (32.2) | 2.246 (2.178, 2.316) | 1.060 (1.056, 1.063) | 1.405 (1.368, 1.443) |
| Sergipe | 17,109 (0.3) | 2.040 (2.034, 2.047) | 1.001 (1.001, 1.001) | 0.943 (0.941, 0.945) |
| Tocantins | 45,712 (0.8) | 1.283 (1.264, 1.301) | 1.021 (1.019, 1.022) | 1.418 (1.401, 1.434) |

Note: Model was adjusted for nighttime relative humidity, PC1, PC2, PC3, and PC4.

**sTable 3. Mean of hot night exposures by group and state.**

|  | Average maximum nighttime temperature (°C) | | Hot night excess (°C) | | Hot night duration  (%) | |
| --- | --- | --- | --- | --- | --- | --- |
|  | Case | Control | Case | Control | Case | Control |
| Acre | 25.74 | 25.84 | 254.03 | 264.67 | 51.9 | 52.8 |
| Alagoas | 26.51 | 26.24 | 287.12 | 281.57 | 43 | 42.99 |
| Amapá | 25.77 | 25.68 | 207.41 | 210.61 | 43.84 | 43.89 |
| Amazonas | 26.34 | 26.3 | 264.11 | 257.65 | 59.27 | 58.4 |
| Bahia | 26.87 | 26.97 | 462.18 | 508.71 | 54.77 | 58.3 |
| Ceará | 27.74 | 27.7 | 453.17 | 442.33 | 59.46 | 58.37 |
| Distrito Federal | 23.74 | 23.93 | 345.65 | 367.78 | 45.4 | 47.14 |
| Espírito Santo | 25.37 | 24.67 | 244.39 | 240.15 | 38.32 | 38.16 |
| Goiás | 26.23 | 26.17 | 412.27 | 413.16 | 51.94 | 52.01 |
| Maranhão | 27.55 | 27.21 | 360.88 | 351.6 | 55.76 | 55.48 |
| Mato Grosso | 26.84 | 26.56 | 344.58 | 345 | 53.06 | 54.1 |
| Mato Grosso do Sul | 27.51 | 27.16 | 253.68 | 266.42 | 39.37 | 41.6 |
| Minas Gerais | 25.48 | 24.99 | 411.23 | 408.16 | 52.26 | 52.3 |
| Pará | 26.53 | 26.38 | 304.01 | 304.91 | 53.84 | 54.24 |
| Paraíba | 28 | 28.16 | 549.21 | 572.24 | 58.25 | 58.89 |
| Paraná | 23.68 | 23.41 | 198.29 | 189.82 | 33.07 | 33.21 |
| Pernambuco | 27.76 | 27.69 | 553.81 | 560.68 | 57.7 | 58.35 |
| Piauí | 28.72 | 28.89 | 548.41 | 562.84 | 61.25 | 61.58 |
| Rio de Janeiro | 24.38 | 23.91 | 182.67 | 184.65 | 32.26 | 31.38 |
| Rio Grande do Norte | 28.4 | 28.89 | 421.02 | 472.84 | 55.59 | 59.09 |
| Rio Grande do Sul | 22.47 | 22.42 | 109.55 | 97.32 | 17.78 | 15.74 |
| Rondônia | 26.23 | 25.82 | 248.82 | 237.62 | 51.19 | 50.3 |
| Roraima | 26.59 | 26.43 | 382.99 | 368.78 | 61.81 | 60.41 |
| Santa Catarina | 19.84 | 20.2 | 66.66 | 63.54 | 13.37 | 12.84 |
| São Paulo | 24.65 | 24.55 | 190.62 | 227.51 | 30.91 | 35.5 |
| Sergipe | 26.21 | 25.93 | 228.84 | 213.28 | 39.35 | 37.91 |
| Tocantins | 27.75 | 27.79 | 409.17 | 401.48 | 55.88 | 53.73 |

**sTable 4. Sensitivity analysis**

|  | Average maximum nighttime temperature, ℃ | Hot night excess, 10℃ | Hot night duration, 10% |
| --- | --- | --- | --- |
| Main analysis | 1.86 (1.86, 1.87) | 1.01 (1.01, 1.01) | 1.05 (1.05, 1.05) |
| Approach 1 | 3.59 (3.58, 3.60) | 1.02 (1.02, 1.02) | 1.13 (1.13, 1.13) |
| Approach 2 | 1.19 (1.19, 1.20) | 1.10 (1.10, 1.10) | 1.01 (1.01, 1.01) |
| Approach 3 | 1.71 (1.70, 1.71) | 1.02 (1.01, 1.02) | 1.00 (0.99, 1.00) |
| Approach 4 | 1.72 (1.72, 1.73) | 1.01 (1.01, 1.01) | 0.98 (0.97, 0.98) |
| Approach 5 | 1.27 (1.27, 1.27) | 1.01 (1.01, 1.01) | 1.09 (1.09, 1.09) |
| Approach 6 | 1.86 (1.86, 1.87) | 1.03 (1.03, 1.03) | 1.34 (1.34, 1.35) |

Note: In approach 1, we excluded dengue cases diagnosed on or after January 1, 2020, to avoid the potential impact of COVID-19 outbreak prevention and control measures on dengue incidence. In approach 2, we excluded dengue cases from São Paulo and Minas Gerais, totaling nearly 3 million cases, in order to avoid possible skews in the dataset that could be caused by the disproportionately large national share of dengue cases in these two states. In approach 3, we defined night as the period from 0:00 a.m. to 8:00 a.m. the next morning local time to describe the time when people usually sleep. In approach 4, we included an additional future control by selecting the same calendar day in the following year, in addition to the two past controls on the same day of the previous years. In approach 5, we selected control days by matching on the same day of the week within the same quarter and year as the diagnosis date. In addition, the date of dengue diagnosis and its control dates were separated from each other by 4 weeks. As a result, each case could be matched with 2 or 3 controls. In approach 6, the threshold temperature (Tthr) was alternatively defined as the 95^th^ percentile of minimum nighttime temperatures from 1990 to 2021 in each state. Model was adjusted for nighttime relative humidity, PC1, PC2, PC3, and PC4.


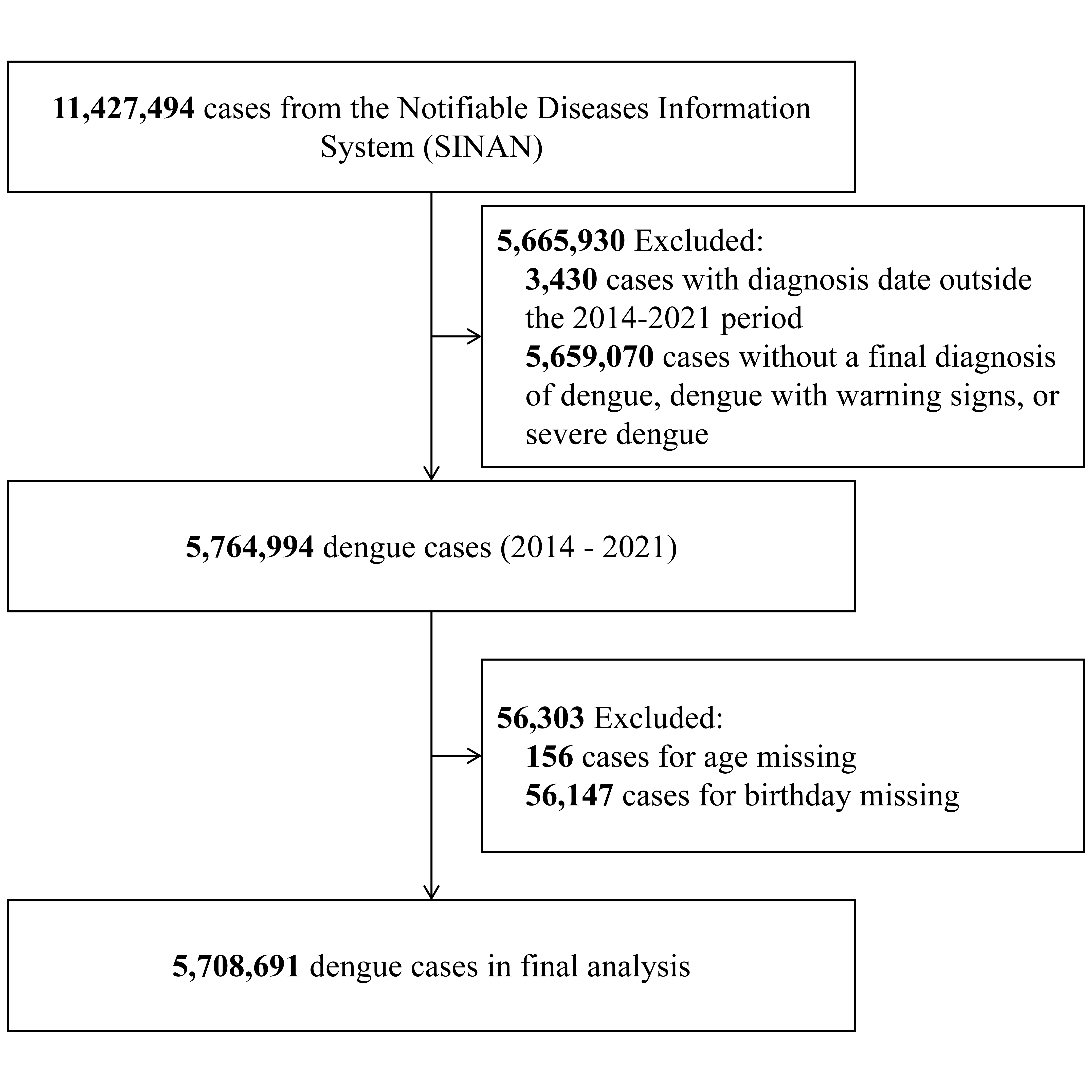


**sFigure 1. The flowchart for the inclusion and exclusion of dengue patients.**

**
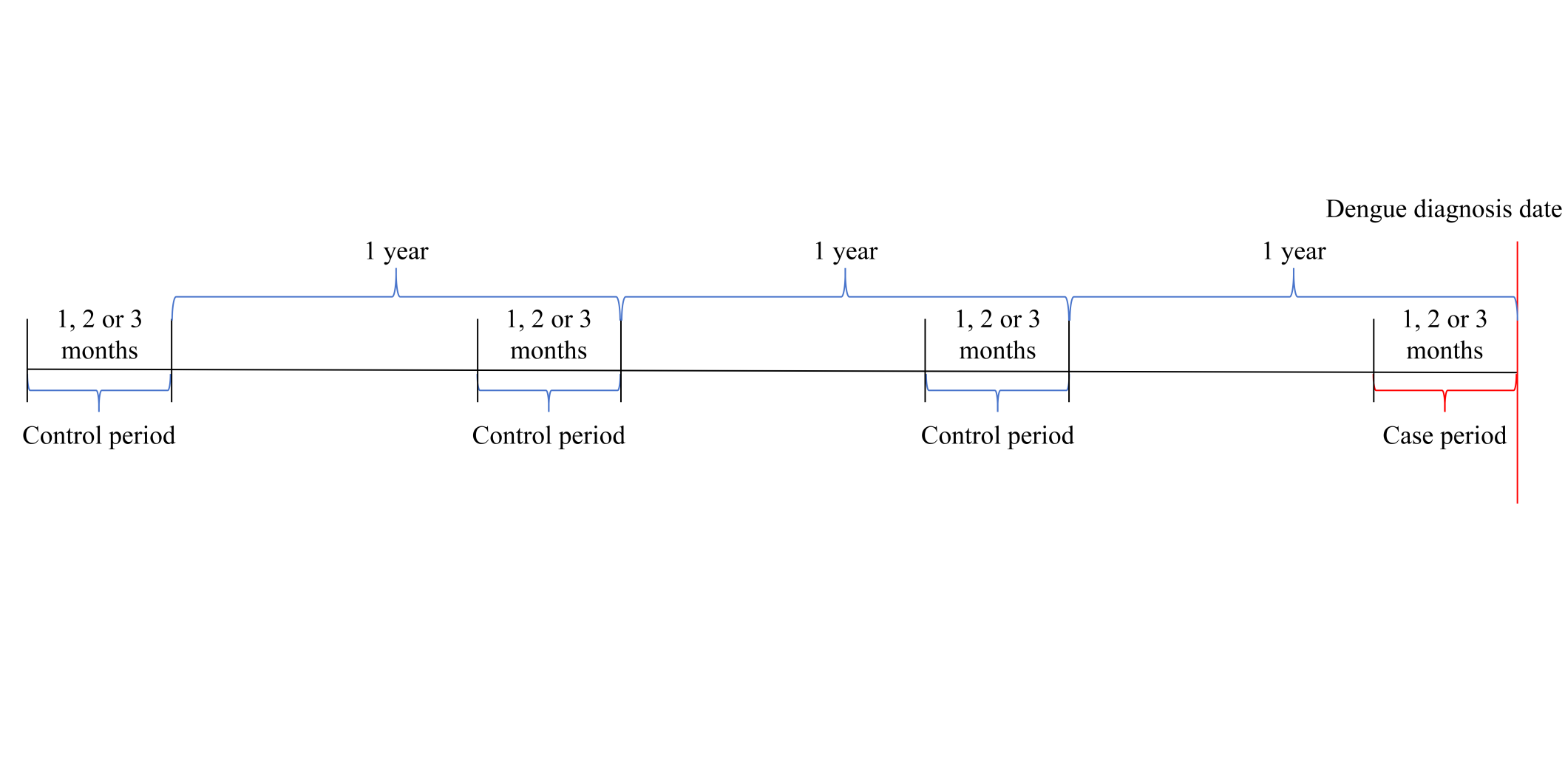
**

**sFigure 2. Case crossover design.**

**
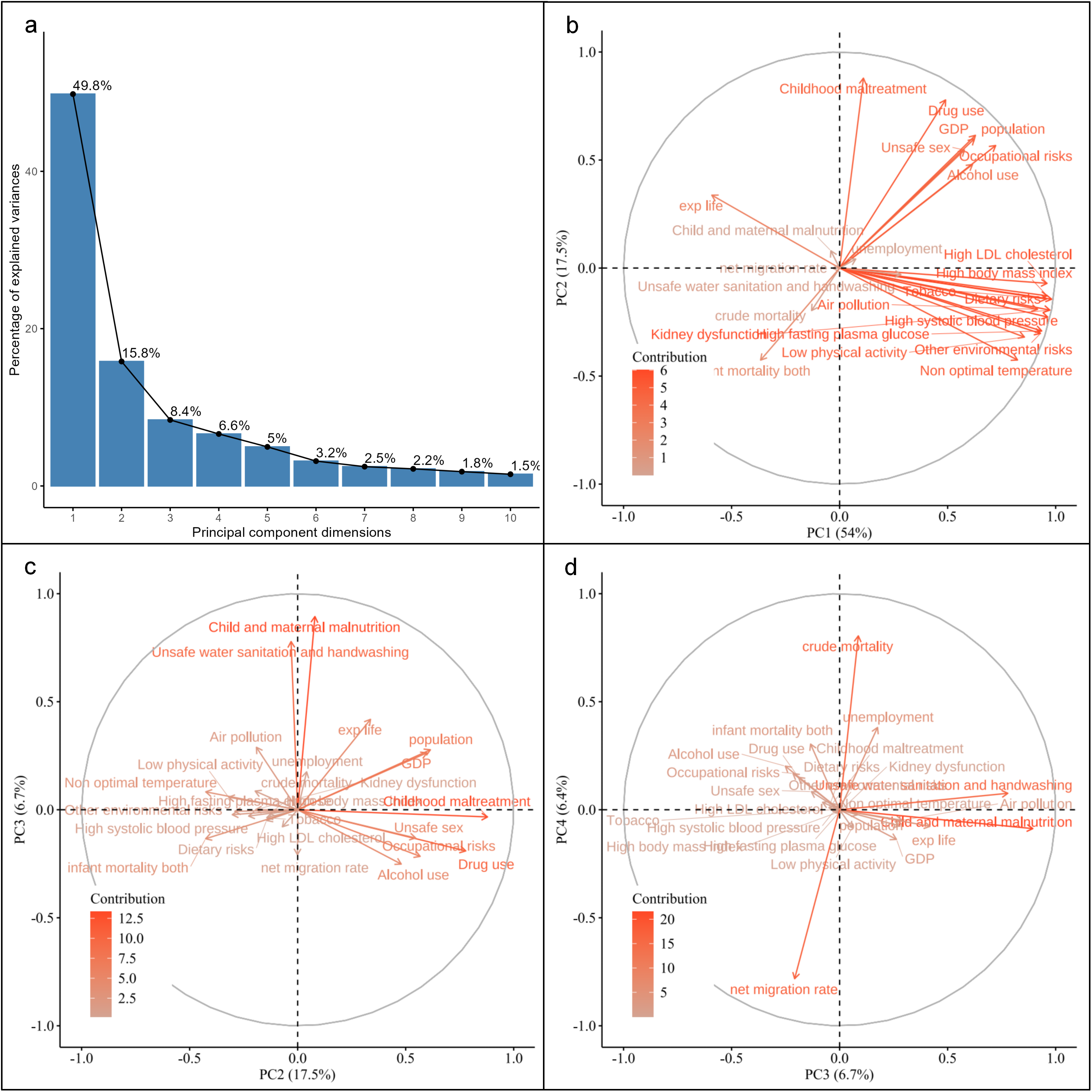
**

**sFigure 3. Principal component analysis of the risk factor and socioeconomic data.**

Abbreviations: PC, Principal component. LDL, low density lipoprotein. GDP, gross domestic product.

Note: (a). Scree plot showing the contribution of the different components to the total variance in the original data the descending order of importance; the four first components explain 80% of the variance. (b, c, d). Contribution of the different risk factor and socioeconomic variables to the four principal axes of variation. Vectors depict the contributions of the variables to two PCs at a time, the first and second in b, the second and third in c, and the third and fourth in d. The colors from light red to dark red indicate the value of the contributions from low to high;


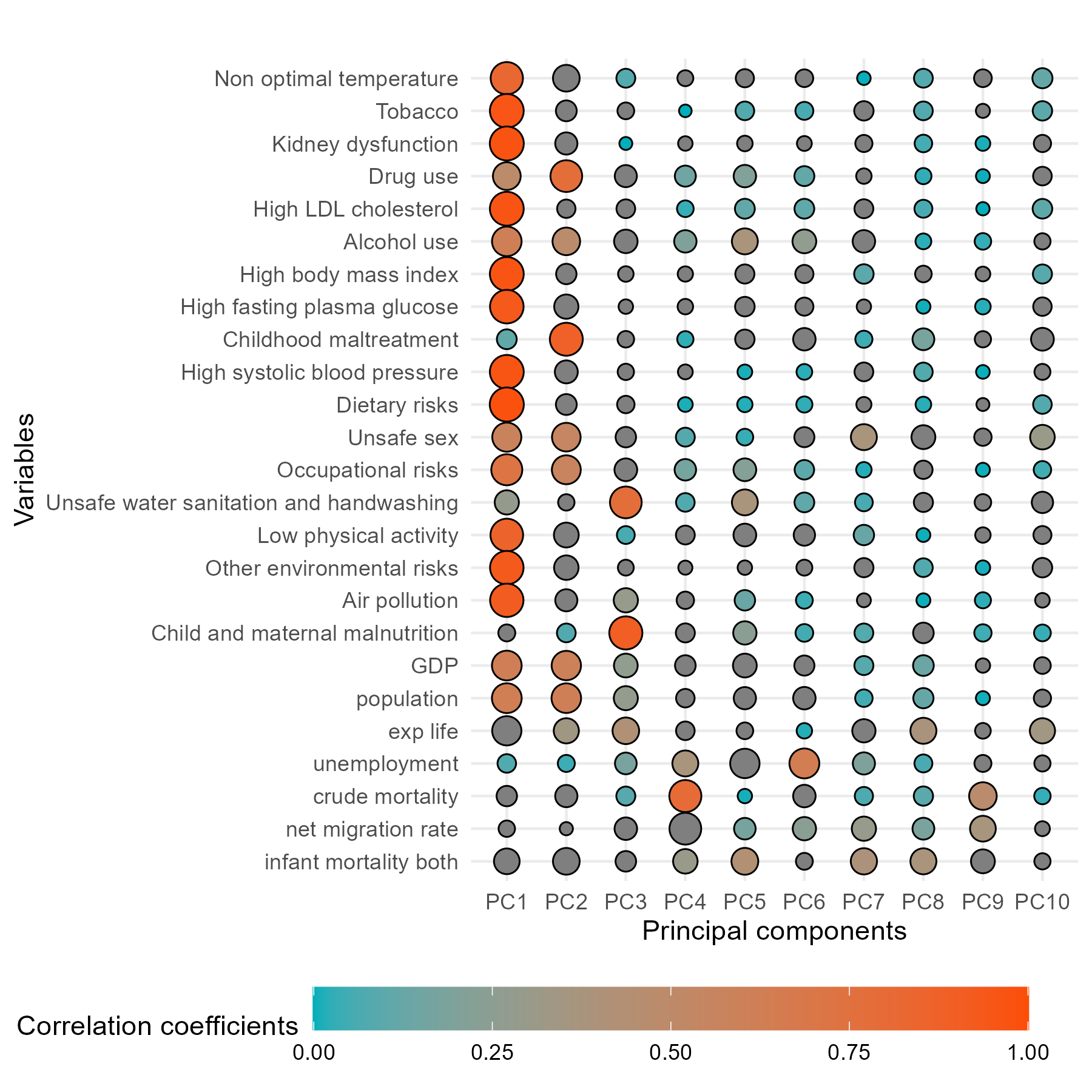


**sFigure 4. Correlation coefficients between the variables and the PCs.**

Abbreviations: PC, Principal component. LDL, low density lipoprotein. GDP, gross domestic product.

Notes: The colors range from blue (representing the lowest correlation coefficients) to orange (representing the highest correlation coefficients), with a smooth gradient in between. The values range from 0 to 1.

**
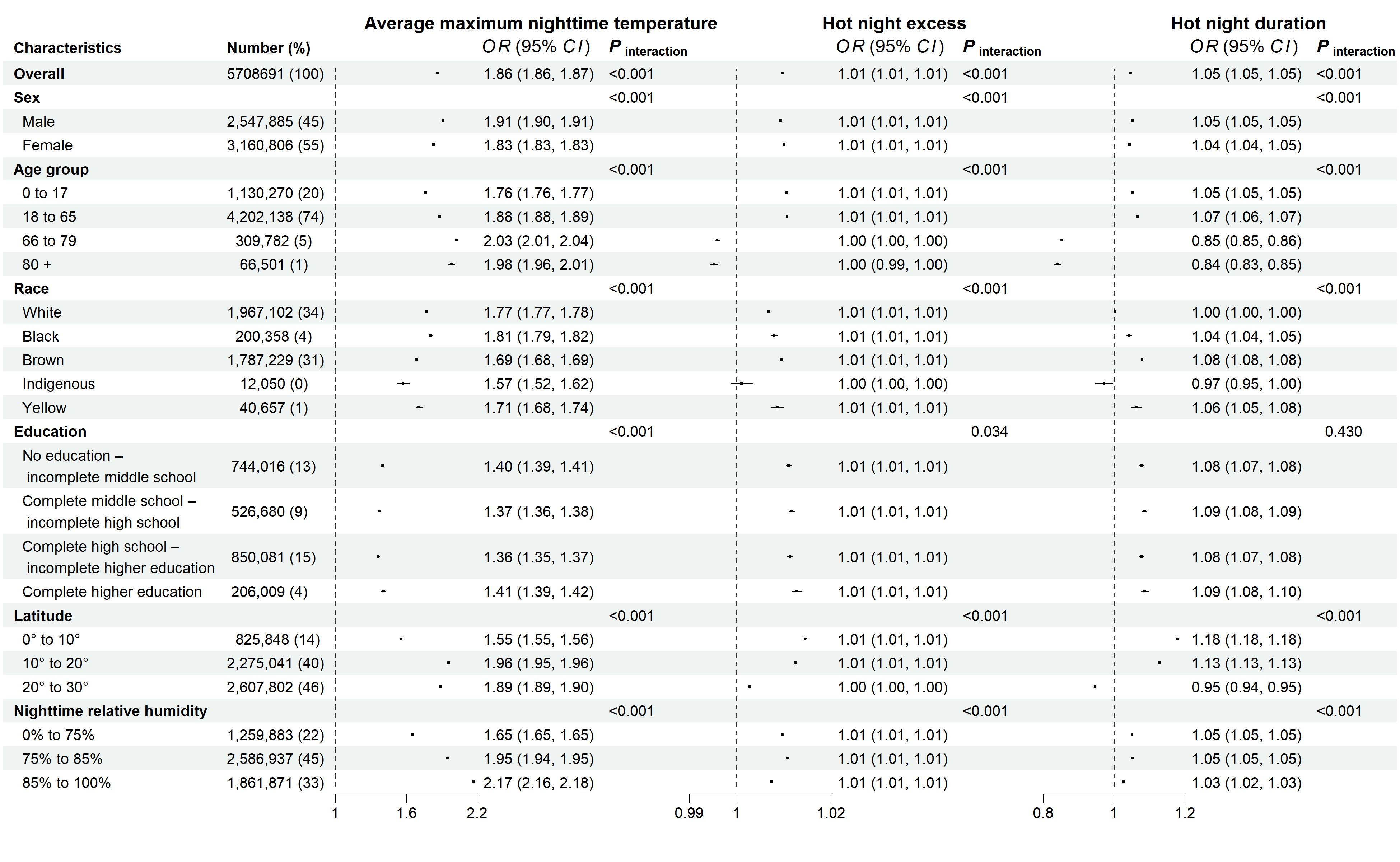
**

**sFigure 5. Subgroup analysis for the association between hot night exposures and dengue incidence.**

Note: Subgroup analyses by sex, age group, and latitude used the entire dataset. Subgroup analyses by race and education used the complete dataset, excluding cases with missing race or education data. The black dots represent the magnitude of the odds ratios (*OR*s), while the horizontal lines crossing the dots indicate the 95% confidence intervals for the corresponding *OR*s.

**
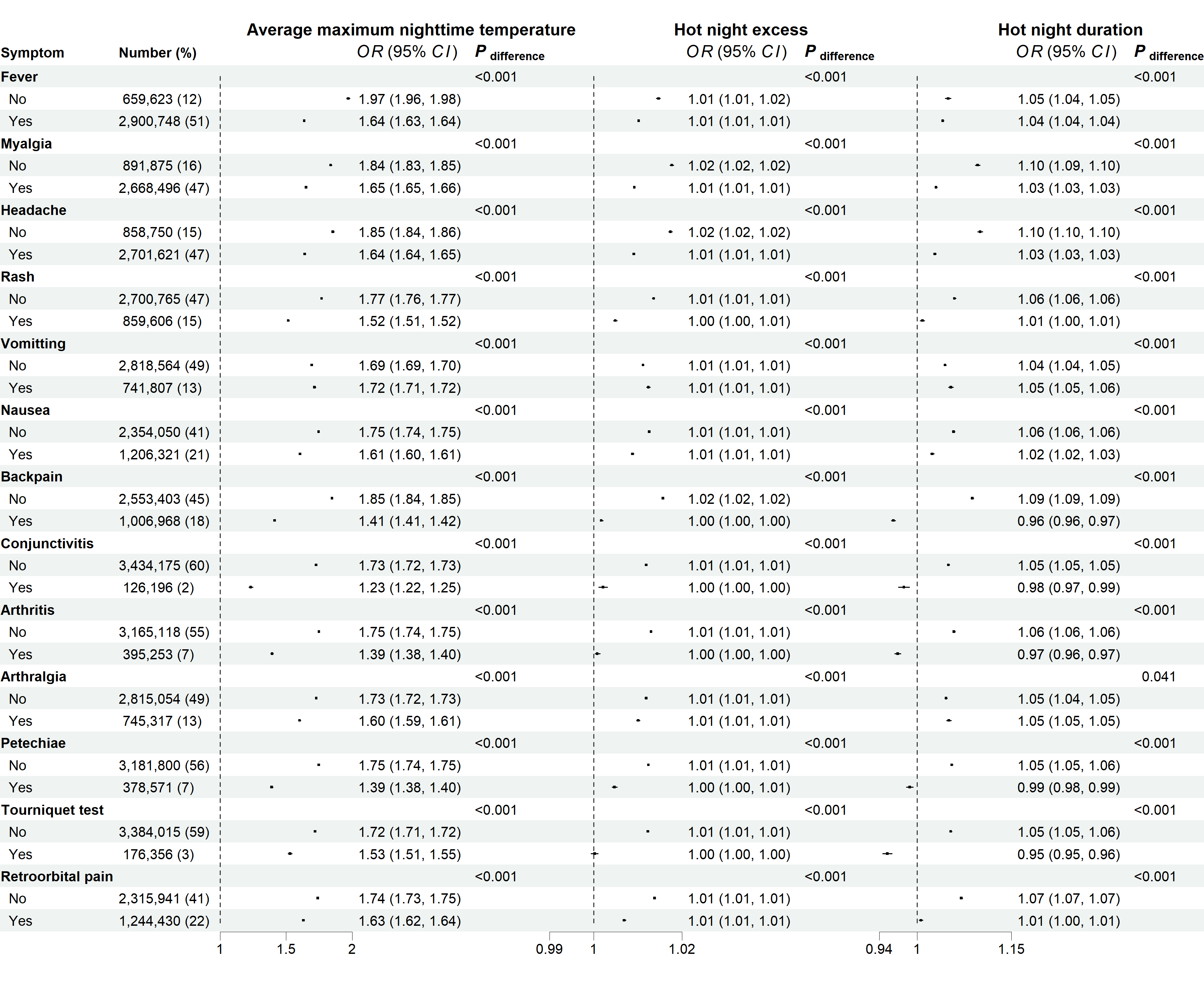
**

**sFigure 6. Odds ratios of hot night exposures on dengue incidence with different clinical symptoms.**

Note: The black dots represent the magnitude of the odds ratios (*OR*s), while the horizontal lines crossing the dots indicate the 95% confidence intervals for the corresponding *OR*s.

**References**

1. Royé D. The effects of hot nights on mortality in Barcelona, Spain. *International journal of biometeorology* 2017;61(12):2127-40. doi: 10.1007/s00484-017-1416-z [published Online First: 2017/08/31]

2. Royé D, Sera F, Tobías A, et al. Effects of Hot Nights on Mortality in Southern Europe. *Epidemiology (Cambridge, Mass)* 2021;32(4):487-98. doi: 10.1097/ede.0000000000001359 [published Online First: 2021/05/04]

3. He C, Kim H, Hashizume M, et al. The effects of night-time warming on mortality burden under future climate change scenarios: a modelling study. *The Lancet Planetary Health* 2022;6(8):e648-e57. doi: 10.1016/S2542-5196(22)00139-5

4. Brown HA, Topham TH, Clark B, et al. Seasonal Heat Acclimatisation in Healthy Adults: A Systematic Review. *Sports medicine (Auckland, NZ)* 2022;52(9):2111-28. doi: 10.1007/s40279-022-01677-0 [published Online First: 2022/04/24]

5. Couper LI, Dodge TO, Hemker JA, et al. Evolutionary adaptation under climate change: Aedes sp. demonstrates potential to adapt to warming. *Proceedings of the National Academy of Sciences of the United States of America* 2025;122(2):e2418199122. doi: 10.1073/pnas.2418199122 [published Online First: 2025/01/08]

6. Lawrence MG. The Relationship between Relative Humidity and the Dewpoint Temperature in Moist Air: A Simple Conversion and Applications. *Bulletin of the American Meteorological Society* 2005;86(2):225-34. doi: <https://doi.org/10.1175/BAMS-86-2-225>

7. Xi D, Liu L, Zhang M, et al. Risk factors associated with heatwave mortality in Chinese adults over 65 years. *Nature Medicine* 2024;30(5):1489-98. doi: 10.1038/s41591-024-02880-4

8. Steenland K, Armstrong B. An overview of methods for calculating the burden of disease due to specific risk factors. *Epidemiology (Cambridge, Mass)* 2006;17(5):512-9. doi: 10.1097/01.ede.0000229155.05644.43 [published Online First: 2006/06/29]
